# Translational stem cell therapy: vascularized skin grafts in skin repair and regeneration

**DOI:** 10.1186/s12967-021-02752-2

**Published:** 2021-02-18

**Authors:** Qian Hua Phua, Hua Alexander Han, Boon-Seng Soh

**Affiliations:** 1 Disease Modeling and Therapeutics Laboratory, A*STAR Institute of Molecular and Cell Biology, 61 Biopolis Drive Proteos, Singapore, 138673 Singapore; 2 Department of Biological Sciences, National University of Singapore, 14 Science Drive 4, Singapore, 117543 Singapore

**Keywords:** Engineered skin graft, Stem cells, Skin regeneration, Vascularization, 3D bioprinting

## Abstract

The skin is made up of a plethora of cells arranged in multiple layers with complex and intricate vascular networks, creating a dynamic microenvironment of cells-to-matrix interactions. With limited donor sites, engineered skin substitute has been in high demand for many therapeutic purposes. Over the years, remarkable progress has occurred in the skin tissue-engineering field to develop skin grafts highly similar to native tissue. However, the major hurdle to successful engraftment is the incorporation of functional vasculature to provide essential nutrients and oxygen supply to the embedded cells. Limitations of traditional tissue engineering have driven the rapid development of vascularized skin tissue production, leading to new technologies such as 3D bioprinting, nano-fabrication and micro-patterning using hydrogel based-scaffold. In particular, the key hope to bioprinting would be the generation of interconnected functional vessels, coupled with the addition of specific cell types to mimic the biological and architectural complexity of the native skin environment. Additionally, stem cells have been gaining interest due to their highly regenerative potential and participation in wound healing. This review briefly summarizes the current cell therapies used in skin regeneration with a focus on the importance of vascularization and recent progress in 3D fabrication approaches to generate vascularized network in the skin tissue graft.

## Introduction

The human body is able to resolve simple wounds naturally over time. However, patients with skin burns or more complex wounds may require extrinsic treatments such as tissue engineered vascular grafts. Autologous grafts are deemed the golden standard for transplantation. However, the risk of infection from the invasive procedure and the lack of donor sites in patients with extensive burns/traumas impose severe limitations. Additionally, traditional cell injections do not maintain essential cell–cell junctions and cell–matrix connections during delivery and the transplanted cells are not restricted to the injured area, thus limiting the efficacy of the treatment [1]. Therefore, tissue engineered vascular grafts serve as the next best alternative. One of the earliest motivations to construct skin tissue stems from the treatment of burn wounds, as ensuring proper wound healing is fundamental to preventing infection. The ability of the skin to heal on its own depends on the depth of the skin injury, which is in turn determined by factors such as the extent of the burns/traumas, intensiveness of the surgeries and genetic abnormalities [2, 3]. Besides therapeutic purposes, there is also an increase in demand for authentic engineered skin equivalents as platforms for drug and formulation development. This review presents a summary on the currently available skin substitutes, with an added emphasis on the significance of the vascularization of skin grafts, as well as recent advances in 3D fabrication techniques to overcome some of the limitations of the current skin substitutes.

### Limitations of commercially available skin substitutes

**Fig. 1:**
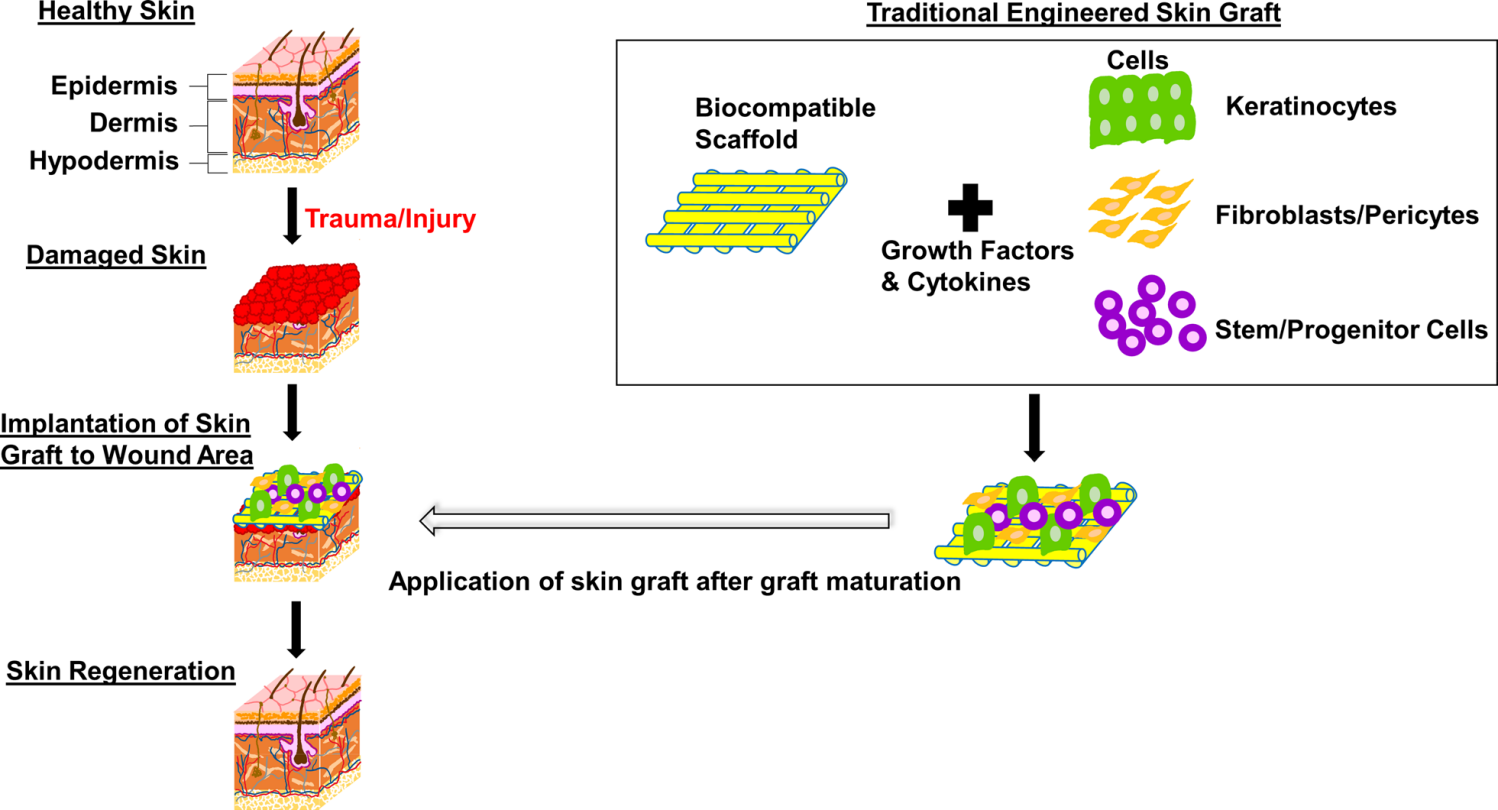
A staged schematic of a traditional engineered skin graft implanted onto the injured skin. Autologous cells are first isolated from the patient’s biopsies and cultured to the desired confluency in vitro. The cells are then seeded onto a biocompatible scaffold in the presence of growth factors and cytokines that aid in the creation and maturation of a functional skin graft. The graft is subsequently implanted onto the wound area to aid in recovery and regeneration of the skin tissue

An extensive loss in skin structure and function may result in serious or potentially lethal conditions that necessitate the use of skin grafts (Fig. 1). The table below summarizes some of the current engineered skin substitutes available clinically (Table 1). Current commercially available skin substitutes are not impeccable and the choice of skin substitutes may be curtailed by factors such as immune rejection, integration failure, high cost, limited cell sources and material incompatibility that impairs successful long-term engraftment [4, 5]. For instance, Biobrane^®^ allows for a single-stage procedure to cover partial thickness wounds, but it is highly susceptible to contaminated wounds [6]. The use of Integra^®^ is likely to result in a good, long-term functional outcome, however, it is highly expensive, displays poor adhesion and is very prone to infection [6].

**Table 1:** List of commercially available skin grafts

Substitute type	Product	Company	Application	Scaffold material	References
Synthetic substitute (acellular)	Integra DRT^®^ (acellular dermal analog)	Integra^®^ Lifesciences Corp., USA	Full-thickness burn wounds, full-thickness non-thermal treatment, chronic ulcer	Cross-linked matrix made up of bovine type-1 collagen and shark chondroitin-6-sulfate coated with silicone on one side	[13–16]
	Alloderm^®^ (human acellular dermal matrix)	LifeCell Inc., Branchburg, NJ, USA	Deep partial and full- thickness wounds	Freeze dried human acellular dermis	[17, 18]
	Biobrane^®^ (epidermis and dermis analog)	Mylan Bertek Pharmaceuticals, USA	Commonly used in pediatric burns and donor sites of split thickness skin	Silicone bonded to woven nylon containing type I collagen peptides	[16, 19]
	OASIS wound matrix	Cook Biotech Inc., West Lafayette, IN, USA	Chronic leg ulcer, acute, chronic and burn wounds	Porcine small-intestine submucosa	[16, 20]
	Hyalomatrix^®^ tissue reconstruction matrix	Fidia Advanced Biopolymers, Abano Terme, Italy	Pressure ulcers, diabetic foot ulcers, and deep second-degree burns	Derivatized hyaluronic acid	[21]
	Matriderm^®^	MedSkin Solution Dr. Suwelack AG, Billerbeck, Germany	Full thickness burns and reconstuctive surgery	Bovine collagen and elastin	[21, 22]
Synthetic substitute (allogenic cells)	TransCyte^®^ (allogenic cellular allogenic skin sub)	Advanced BioHealing, Inc., USA	Partial-thickness burn wounds and donor site treatment	Neonatal allogenic fibroblast cultured on collagen-coated nylon mesh	[16, 23]
Natural skin substitute (allogenic cells)	Dermagraft^®^ (allogenic dermal sub)	Advanced BioHealing, Inc., USA	Chronic wounds and diabetic foot ulcers	Biodegradable polyglactin mesh combined with neonatal foreskin fibroblast	[24, 25]
	Apligraf^®^ (allogenic—both living epidermis and dermis)	Organogenesis Inc., USA	Diabetic foot ulcers	Bovine type I collagen matrix combined with allogenic neonatal foreskin fibroblast and keratinocytes	[26]
	Orcel^®^ (allogenic—both living epidermis and dermis)	Ortec International Inc., New York, NY, USA	Treat donor sites in burns, surgical wounds, partial thickness wounds and recessive dystrophic epidermolysis bullosa	Bovine collagen type I matrix seeded with fibroblast and keratinocytes derived from neonatal foreskin	[27, 28]
Natural skin substitute (autologous cells)	Epicel^®^	Genzyme Biosurgery, USA	Full thickness burns	Autologous keratinocytes sheets attached to petrolatum gauze support	[29, 30]
	Epidex	Modex Therapeutiques, Lausanna, Switzerland	Improvement of relatively small chronic leg ulcers	Cultured keratinocytes sheets derived from outer root sheath of anagen hair follicles	[28, 31]
	Laserskin^®^	Fidia Advanced Biopolymers Srl, Italy	Foot ulcers	Autologous keratinocytes and fibroblasts, grown on microperforated hyaluronic acid membranes	[6, 16]

Till date, there is no available engineered skin tissue able to permanently cover full thickness or deep dermal wounds in a one-stage procedure. Additionally, most of the skin substitutes available include only fibroblast and keratinocytes, which are insufficient in recapitulating the complexity of native skin [6]. Scar formation following skin grafting also impairs the functionality of the skin. Since scar tissue is less resistant to UV radiation, the skin has difficulty repopulating hair follicles and sweat glands [7]. Lastly, the lack of early and proper vascularization leads to graft necrosis or loosening of implanted skin substitutes, ultimately culminating in poor skin engraftment [8, 9]. Several papers have explored the possible inclusion of a hypodermal layer within the skin construct that may contribute to graft viability [10, 11]. Since the hypodermis primarily consists of endothelial cells (ECs) and adipocytes, the presence of such a layer in the graft provides thermoregulation and structural support against mechanical insults, as well as nutrients and signaling cues for vessel formation [5]. However, further studies are required to ascertain the clinical efficacy of a hypodermis-inclusive 3-layered skin graft [5]. Commercially available skin substitutes that promote angiogenesis do not attain the magnitudes required for good engraftment. Hence, the vascularization of skin grafts remains the top issue to be addressed in developing the ideal functional skin substitute for clinical applications [12].

### The process of vascularization

**Fig. 2:**
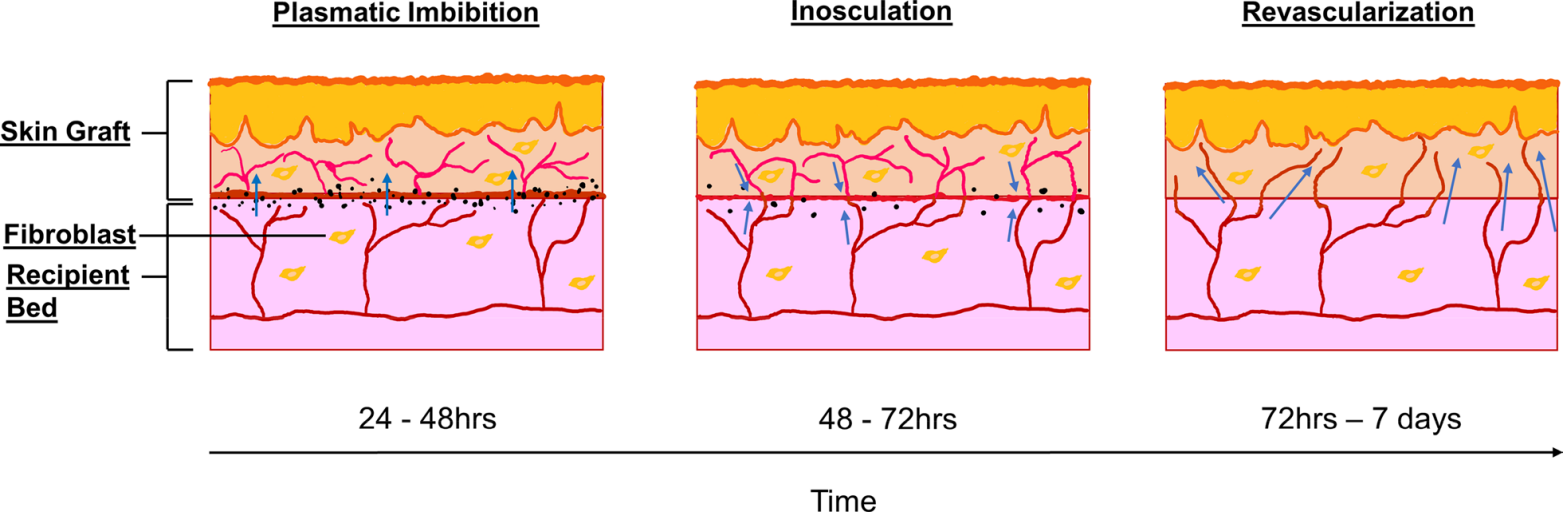
A schematic representation of the process of vasculogenesis and angiogenesis subsequent to the skin graft transplantation. In the early stages following transplantation, nutrients from the wound bed diffuse along a gradient into the graft via the process of plasmatic imbibition but is limited by the range of diffusion. Around 48 h after the transplantation, the vessels of the host tissue begin to form anastomoses with the vascular buds of the graft during inosculation. The inception of a functional vascular network between the graft and the recipient bed enhances the survivability of the graft. The process of revascularization occurs within 72 h whereby the ingrowth of new vessels from the recipient bed to the graft, accumulation of extracellular matrix and the subsequent maturation of the new vessels augments the stability of the engrafted tissue

Vascularization is a complex process orchestrated by the combination of various cell types, growth factors and extracellular matrix proteins. Vascularization in vivo comprises of two distinct mechanisms; vasculogenesis and angiogenesis [32]. During vasculogenesis, the endothelial progenitor cells migrate, differentiate and form primitive blood vessels, whereas angiogenesis involves the growth of new vessels from current pre-existing ones (see Fig. 2) [33]. There are two approaches to incorporating vessels in the engineered tissue, either in vitro before transplantation or vascularization upon integration with host tissue [34]. Importantly, the micro-vascular network generated has to exhibit a functional and sustainable phenotype during both in vitro development and post-implantation in vivo even when the original conditions were absent. To produce mature and stable micro-vessels, the ECs proliferate and form capillaries through specific cell-to-cell contact, dictated by the release of growth factors in a specific chronological order [35].

During the wound healing process, vascular smooth muscle cells (VSMCs) initiate vessel formation by recruiting ECs to the site of angiogenesis and promoting EC proliferation through vascular endothelial growth factor (VEGF) secretions [36, 37]. On the other hand, fibroblast growth factor (FGF) increases EC migration and enhances VEGF production, highlighting the importance of cross-talk between growth factors. Some of the common growth factors and cytokines required for angiogenesis include VEGF, FGF, tumor necrosis factor-alpha (TNF-$\mathrm{\alpha })$, transforming growth factor (TGF-$\upbeta$) and angiopoietins [38].

The angiogenic signaling pathways mediated through growth factors have been well documented [39]. More recently, ECs have been shown to respond to metabolic changes that switch the cells from a quiescent state to a decidedly proliferative profile, consequentially driving the process of vessel sprouting [40]. To satisfy the higher energy demands during the angiogenic state, ECs upregulate the rate of aerobic glycolysis to generate more ATP [41]. This process is aided by angiogenic factors such as VEGF that can enhance the expression levels of glycolytic activators in ECs [42]. Given that the major source of ATP in the ECs is derived from glycolysis instead of oxidative phosphorylation [42], the role of the endothelial mitochondrion as an ATP generator is somewhat diminished when compared to other cell types [43]. However, the endothelial mitochondria retain the propensity for the cells to switch over to oxidative metabolism when the levels of glucose are significantly reduced [43]. Through the tricarboxylic acid (TCA) cycle, the mitochondria generate precursors that are crucial for the synthesis of nucleotides, lipids and amino acids during cell proliferation [41, 43]. In addition, physiological levels of reactive oxygen species (ROS) produced by the mitochondria as a by-product of oxidative phosphorylation serve as signaling cues for the initiation of angiogenic cascades [44]. For example, mitochondrial ROS had been shown to increase the stability of hypoxia-inducible factor α through the inhibition of prolyl hydroxylase domain proteins, resulting in the activation of angiogenic pathways [41, 45].

In pathological states such as diabetes where patients are afflicted by chronic wounds, lowering the excessive levels of mitochondrial ROS is vital to the resolution of the skin wound [46]. Pre-clinical studies have shown that a mitochondria-targeted antioxidant, SkQ1, was able to resolve the inflammatory phase of wound healing, leading to vascularization and improvements in the dermal wounds of genetically diabetic mice [47]. There is also an on-going clinical trial investigating the use of antioxidant-laced dressing derived from locust bean gum galactomannan, curcumin and *N*-acetylcysteine in treating chronic wounds [48].

ECs and SMCs are the major cell types in the vessels. However pericytes, together with FGF and collagen IV are also required for the maturation and stabilization of the blood vessels, regulation of angiogenesis, and inhibition of uncontrolled growth [49]. Pericytes regulate vessel blood flow through their contractile properties and provide supportive function by laying down basal extracellular matrix (ECM) [50]. Apart from playing a role in mediating EC-pericyte interaction, TGF-$\upbeta$ also aid in stabilizing the vessels [38].

### Vascularization in engineered skin tissue

Proper vascularization of the engineered skin tissue is salient when constructing a functional replacement to the damaged skin. Full ingrowth of blood vessels is crucial in supplying the embedded cells of the engineered tissue with oxygen and nutrients. Without the vessels to promote proper diffusion of oxygen and nutrients, cells may lose their functionality and die from hypoxia [51, 52]. Furthermore, the vessels allow for the efflux of carbon dioxide and cellular waste products. Previous studies have shown that ECs alone are inadequate in forming self-sustainable and sturdy vessel networks [37, 50, 53]. Co-culturing ECs with supportive cells such as vascular smooth muscle cells, pericytes and fibroblasts are essential to the vessel construct [37, 50, 53]. Scientists have managed to construct vessels made of human umbilical vein ECs co-cultured with fibroblasts, that successfully incorporated into the dermal layer in vitro [53].

Vascularization also plays a role in graft innervation, with multiple studies demonstrating that neovascularization occurs before nerve innervation [54, 55]. Hobson et al*.* reported that in well-vascularized areas with longitudinally oriented vessels, regeneration of Schwann cells and axons were the highest [34]. Interestingly, a recent study reported that ECs embedded in microvascularized tissue in vitro guided neuronal precursors through the secretion of brain-derived neurotrophic factor [56]. Collectively, these studies demonstrated the importance of well-vascularized tissue construct in nerve regeneration and recovery.

### Design components of an engineered skin graft

There are several factors to consider during the construction of artificial skin tissue. Firstly, the types of cell to be used and the sources which these cells are obtained from is crucial. Proliferative cell populations can be isolated from biopsies and cultured in vitro [6]. Alternatively, a self-renewing pool of iPSCs derived from the patients can differentiated into the desired cell types indefinitely [6]. Equally important is the selection of a suitable biopolymer that can be developed into a 3D scaffold, allowing the cells to anchor and seed properly. The skin construct is then allowed to mature in the presence of growth factors and cytokines which aid in cell proliferation and vascular development.

#### Cell sources and growth factors in engineered vascularized skin tissue

The selection of the optimal cell source is vital in developing the engineered tissue. Allogenous ECs are very immunoreactive, hence less suitable for the purpose of skin grafts [34]. Alternative cell sources such as autologous differentiated cells and stem cells have been experimented to construct the skin tissue [57]. While differentiated cells such as keratinocytes and fibroblasts are more physiologically similar to the endogenous cell populations, their low proliferative capacity requires a greater number of cells to be seeded [57]. This is especially true for larger skin grafts. Additionally, the explant procedure of vascular ECs from saphenous vein is highly invasive, whereas only a small number of microvascular ECs can be harvested from skin biopsies [34]. Therefore, the utilization of iPSC-derived ECs and VSMCs in the construct of engineered vascularized skin tissues has been explored to avoid the shortcomings of primary cell types. iPSCs exposed to PDGF and VEGF in vitro were able to differentiate successfully into functional VSMCs and ECs with similar properties to endogenous vascular cells [58]. Additionally, Stebbins et al*.* showed that co-culturing of iPSC-ECs and iPSC-derived pericytes resulted in organized tube-like structures by day 7 [59].

Similarly, mesenchymal stem cells (MSCs) can be directed to differentiate along the endothelial cell lineage [60]. MSCs also aid in facilitating the maintenance of vessels through the secretion of various growth factors such as VEGF, HGF, IGF-1 during vascularization [61]. Taken together, stem cells have demonstrated their potential in generating essential vascular cells and growth factors to be used for vessel development. Recent developments in nanomedicine also offer alternative strategies in managing chronic wounds. Nanoparticles carrying growth factors and antibiotics can be formulated as slow-releasing molecules that enhance the bioavailability of the therapeutics [62, 63]. Similarly, nanomaterials that mimic the native structure of the tissue can facilitate the attachment of fibroblasts, keratinocytes and ECs to the skin graft, thereby enhancing re-epithelialization and angiogenesis [62, 63].

Selecting an optimal biomaterial is critical to the graft construct. This was demonstrated in other studies where the use of nanomaterials play crucial role in tissue engineering for cartilage regeneration [64]. Multiple factors such as biodegradability, biocompatibility and mechanical properties have to be taken into consideration [57]. However, the discussion of this topic is beyond the scope of the current review and has also been extensively discussed recently [57, 64, 65].

#### Pre-vascularized graft

Developing a pre-vascularized graft promotes integration with the host vasculature and improves the viability rate of the skin construct, hence attaining a higher success rate of implantation [66]. Chen et al*.* demonstrated that pre-vascularized human MSC sheets, transplanted with autologous split thickness skin graft, aid to accelerate wound healing in rat full thickness skin wound models [1]. Pre-formed vessels allowed for essential nutrients and growth factors to be delivered to the cells on the cell sheet, thus promoting survival of these cells and enhance the incorporation into the host tissue [1]. A more recent study conducted by Miyazaki et al*.* demonstrated promising results of expedited wound healing by implanting a novel 3D scaffold-free pre-vascularized substitute onto immuno-deficient mouse models [12]. While non-vascularized tissue constructs showed poor collagen deposition possibly due to the poor blood supply, epidermal sloughing was not observed in pre-vascularized substitute [12]. Therefore, a greater number of vessels is evidently critical for the enhanced maintenance and survival of the graft, especially in the initial phase. Interestingly, 7 days after grafting, significant differences were observed. Blood vessels were found only on the circumferential border of the non-vascularized graft, whereas blood vessels found in pre-vascularized graft was well-perfused [12]. Besides good graft adherence, pre-vascularized skin substitute permits rapid perfusion which enhances the deposition of collagen and increased dermal thickness [12]. The results mentioned by the study illustrated the benefits and importance of having a pre-vascularized tissue substitute.

Pre-vascularization of the engineered skin tissue induces a high level of neovascularization that is crucial in ensuring the survival of the transplanted grafts [1]. Skin sheets which are pre-vascularized also enhance the healing of full thickness skin wounds through elevated angiogenic factors that mediate paracrine signaling during the wound healing process [1]. Angiogenesis is vital in supplying nutrition and oxygen through the new vessels to support cells at the damaged site. Enhanced angiogenesis observed in pre-vascularized grafts would therefore be more likely to expedite skin rejuvenation. In additional, pre-vascularized scaffolds have been reported to integrate better with the host vasculature [36].

#### 3D bio-fabrication of pre-vascularized engineered skin tissue

In the past, 2D cell cultures of keratinocytes were used to study and model the pathology of various skin diseases, which imposes constraints on accurate responses to drugs due to the morphology of the cells being different as the cell culture condition contains a single cell type population only [67]. Simple manual fabrication techniques such as manual dispensing, molding and freeze drying would have its limitations in recapitulating the architecturally complex vasculature of the native tissue [68].

However, a 3D cell culture system creates a native in vivo-like environment, which is especially important for accurate skin modelling. By mimicking the environment, it also permits the cells to fully recapitulate the morphology of keratinocytes through the different cell–cell synergistic interactions [69]. While there are many existing commercially available skin constructs, the constructs are still lacking in many aspects, such as poor vascularization and missing hair follicles [70]. With frontiers constantly pushed, the field of 3D culture has evolved rapidly and current bioprinting approaches are automated to fabricate customizable constructs incorporating heterogenous population of cells, alongside suitable biomaterials as scaffolds to stimulate and mimic the complex nature of the native skin so as to improve the functionality of the construct.

#### Approaches to constructing the vasculature

**Fig. 3:**
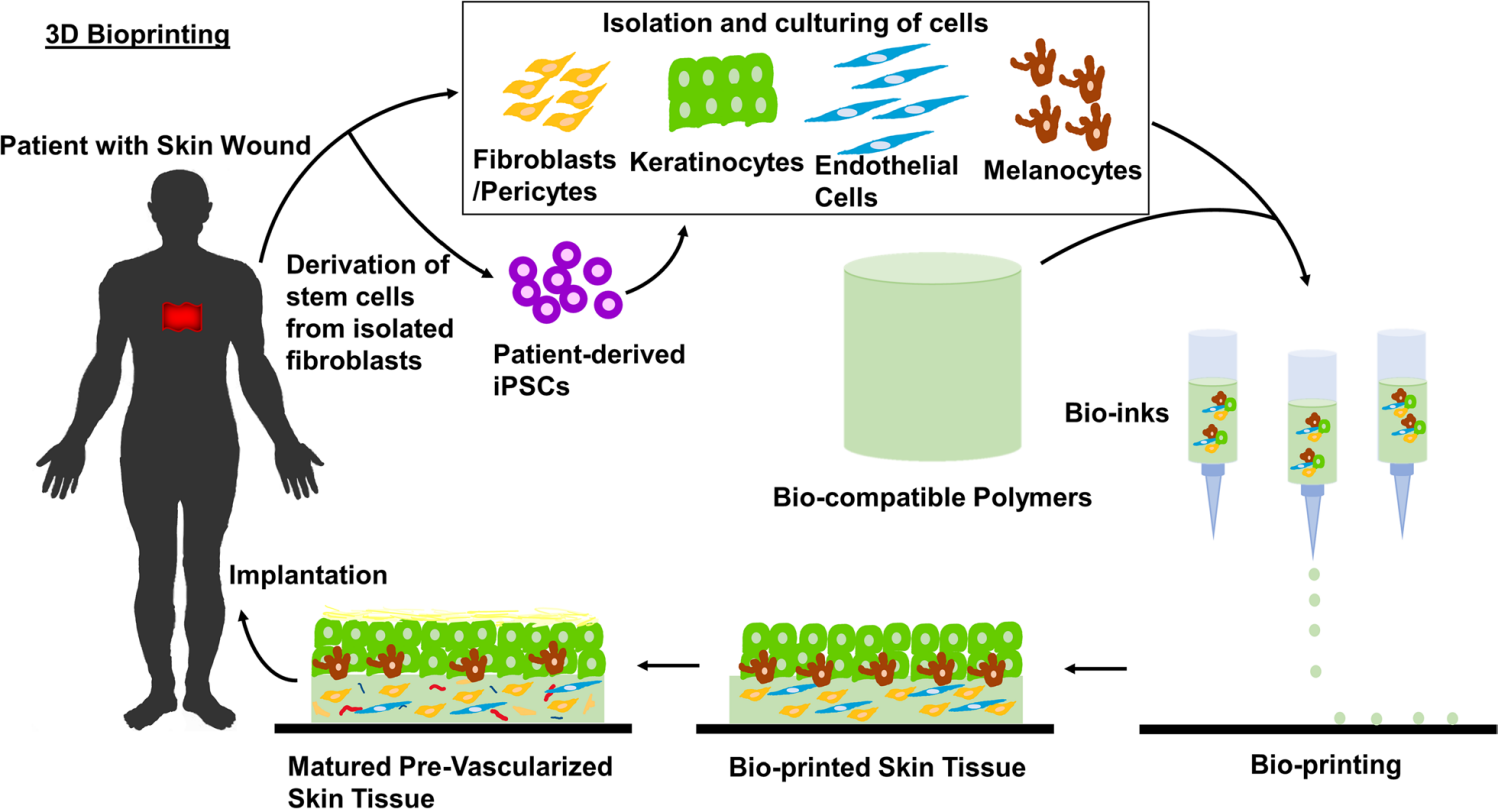
A schematic representation of the 3D bioprinting of engineered skin tissue. Cells are isolated from the patient’s biopsy and cultured in vitro to reach sufficient numbers. Isolated fibroblasts can also be re-programmed into iPSCs, which can subsequently be differentiated into the desired cell types and amounts within a shorter period of time. Bio-inks usually comprise of live cells encapsulated by a biopolymer material which acts as a scaffold. Using 3D technology, skin tissues are bio-printed in a conformationally defined manner and allowed to mature before implanting the tissue onto the patient

3D bio-fabrication techniques such as 3D bioprinting, micro-patterning using hydrogel based-scaffold, nano-fabrication and mechanical spacers have been developed to generate the required vasculature network [71–73]. The technology of 3D bioprinting incorporates microvasculature within the tissue construct which enables anastomosis to occur with the host blood vessels for better perfusion of blood supply (see Fig. 3) [34]. Bioprinting is a method which uses bioink, a hydrogel made up of multiple materials, ranging from synthetic biomaterials (pluronic and poly(ethylene glycol)) to natural biomaterials (agarose, gelatin and alginate) to print a variety of structures which support vascularization, in in vitro models [74]. During the bioprinting process, the cell-compatible bioinks will cross link, to form a stable hollow construct, providing mechanical support to the cells while supporting cellular processes underlying vascularization [75]. Besides printing the microvessels, it is also important to ensure that the vessels are perfusable.

Recently, there have been reports of successful fabrication of perfusable skin constructs with similar biological properties to native skin 3D printing technology. Baltazar et al*.* bioprinted vascularized skin grafts through using bioinks containing a heterogenous cocktail of cells including human foreskin dermal fibroblasts, endothelial cells, placental pericytes and foreskin keratinocytes [76]. The bioprinted constructs are then subsequently submerged in endothelial growth medium to allow self-assembling of vascular network [76]. While the keratinocytes proliferate and mature to form the multilayered skin construct in vitro, endothelial cells and pericytes form endothelial network [76]. Besides providing extra stability to the microvessels, interestingly, pericytes were found to also enhance maturation of the keratinocytes, producing a more ordered stratification and thickened epidermis [76]. When the vascularized skin graft was implanted onto the immunodeficient mouse, the skin graft was perfused and the microvessels remain present 4 weeks post-engraftment [76]. Harnessing both stem cell and 3D printing technology, Abaci et al*.* micropatterned perfusable vascular network skin construct containing iPSC-derived keratinocytes, melanocytes, fibroblasts and endothelial cells [66]. 3D printing technology were utilized to construct the desired mold patterns composing of alginate to create the microchannels [66]. Subsequently, to test the functionality, the vascularized skin construct was grafted onto the back of SCID mice and demonstrated neovascularization guidance during wound healing, which may improve the integration of the skin construct [66]. Amongst all the techniques, 3D bioprinting has been the most extensively used as the complex vasculature network can be printed out with profound precision and flexibility. The 3D structure is created via impeccable layer-by-layer deposition of materials in a desired pattern through advanced computing [77]. There are three types of 3D bioprinting techniques; ink-jet, laser-based and extrusion, each offering stellar results in generating intricate vasculature [78].

In ink-jet printing, cell-incorporated alginate in the form of hydrogel is used as a scaffold with calcium chloride as a cross-linker. The inkjet printers allow for multiple cell types to be deposited onto the scaffold in a controlled and organized computer-aided design [79]. A study reported on the incorporation of three bioinks, constituting canine smooth muscle cells, bovine aortic endothelial cells and human amniotic fluid-derived stem cells, onto an alginate-collagen scaffold by a thermal inkjet printer. The tissue was functional and vascularized when the scaffold was implanted in vivo [80].

Laser-based bioprinting has been explored in both 2D and 3D patterning at high resolution without nozzle clogging. Using this printing technique, human umbilical vein endothelial cells (HUVECs) with hMSCs had been printed to create a cardiac patch to be implanted on a myocardial infarcted rat heart. The patch enhanced capillaries formation and improved the function of the infarcted heart [81].

Lastly, extrusion-based bioprinters with a coaxial nozzle have the ability to print hollow filaments with microfluidic channels that form the vascular network. Luo et al*.* reported hollow alginate-poly(vinyl alcohol)fibers, created from the core nozzle of extrusion-based bioprinters, possessing sufficient mechanical strength to support human bone marrow stem cells attachment and spreading [82]. Altogether, bioprinting offers the possibility to incorporate desired cells (at an optimal density) onto a specially computer-designed biocompatible scaffold with vessels-like channels in a near-native microenvironment.

In 2014, Lee et al*.* bioprinted keratinocytes, fibroblasts and collagen to represent the native human epidermis, dermis and dermal matrix, respectively [83]. Histology and immunofluorescence characterization proved that the 3D printed skin tissue was morphologically and biologically representative of in vivo human skin tissue [83]. Subsequently, a hybrid 3D cell-printing system combining inkjet and extrusion modules was developed to generate a human skin model with a functional transwell system [84]. The extrusion module was used to generate the collagen-based construct with polycaprolactone (PCL) mesh, while the keratinocytes were deposited with inkjet modules. The skin model was able to mature into stabilized epidermis and dermis after 14 days [84].

### Challenges

One technical challenge to be addressed during the creation of microvascular networks in engineered tissue is to ensure that the network vessels stay stable during the production at the in vitro level and remain functional when it is implanted onto the injured area. The process of vessels generation is a complex procedure, besides EC proliferation, a plethora of growth factors has to be delivered in a sequential manner, within certain time frame to ensure cell–cell contacts during the formation of interconnected microvessels network [35]. As such, 3D printing has the advantage of enabling predefined deposition of essential factors in a timely manner at particular concentrations. To effectively translate the use of 3D bioprinted skin for downstream clinical applications, there has to be a comprehensive system set up to monitor the quality of each step of the bioprinting process. These includes the quality of the scaffold to avoid the risk of contamination as well as ensuring that the viability of the cells stay high [85]. All in all, a robust protocol has to be in place for a successful bench to bedside translation. While 3D bioprinting provides the luxury of ensuring robustness in printing customizable grafts, reproducing the function of skin such as thermoregulation, perspiration and sensation serves as key limitations. Additionally, till date, most of the implants have only been conducted on rodent models, which have smaller wound areas. Hence, in the future, skin grafts on larger wound area will have to be tested for effective bench to bedside translation.

#### Future directions on engineered skin grafts

Proper vascularization is definitely the key element to be addressed for successful grafting. During the initial phase, a well-developed vessel bed is especially important for essential nutrients to reach the metabolically demanding cells to prevent tissue necrosis. In the future, smart scaffolds with tailored biological properties that directs the formation and maintenance of the hierarchal vasculature network through biochemical cues could be the next breakthrough in skin tissue regeneration.

In the current era of regenerative medicine and tissue engineering, the future of dermal regeneration is heavily reliant on stem cells. Banking of autologous iPSCs avoids the tedious process of isolating cells from the patients and allows the cells to be utilized immediately for transplantation or grafting. iPSC banks are also able to ensure the high quality and survival rates of post-thawed cells through extensive characterization during the freezing down process. Currently, there are multiple iPSC banks located globally in Japan, USA, Europe and Taiwan [86]. As more cell banks are established, it is likely that ready-made, off-the-shelf iPSC lines could be available in the future for emergency cases which requires immediate skin grafting.

## Conclusion

Existing skin substitutes currently used in burn wounds and ulcer treatment are unable to recapitulate the native complexities of the skin dynamic and structure. While 3D bioprinting holds great potential in creating functional skin equivalents, further studies have to be conducted to elucidate the choice of scaffold materials that fulfill the biocompatibility and biodegradability requirements. Additionally, a new design process is required to better represent the heterogeneity of the skin construct. Other considerations include a reduction in or no scar formation and integration of functional skin appendages similarly to the local physiological organization, while keeping the wound infection rate low. More importantly, reciprocity of signaling pathways between the scaffold, cells and the growth factors has to be established to support vascularization.

## Data Availability

Not applicable.

## Abbreviations

EC: Endothelial cell
EGM-2: Endothelial growth medium 2
FGF: Fibroblast growth factor
HGF: Hepatocyte growth factor
HUVEC: Human umbilical vein endothelial cell
IGF-1: Insulin-like growth factor-1
iPSC: Induced pluripotent stem cell
MSC: Mesenchymal stem cell
PCL: Polycaprolactone
ROS: Reactive oxygen species
SCID: Severe combined immunodeficient mice
TCA: Tricarboxylic acid
TGF-β: Transforming growth factor-beta
TNF-α: Tumor necrosis factor-alpha
VEGF: Vascular endothelial growth factor
VSMC: Vascular smooth muscle cell
